# Exercise Promotes the Osteoinduction of HA/β-TCP Biomaterials via the Wnt Signaling Pathway

**DOI:** 10.3390/metabo10030090

**Published:** 2020-03-05

**Authors:** Lijia Cheng, Ahmad Taha Khalaf, Tianchang Lin, Ling Ran, Zheng Shi, Jun Wan, Xin Zhou, Liang Zou

**Affiliations:** College of Medicine (College of Nursing), Chengdu University, Chengdu 610106, China; ahmadtaha11@yahoo.com (A.T.K.); tianchanglin1999@outlook.com (T.L.); rldgj@163.com (L.R.); drshiz1002@hotmail.com (Z.S.); wanjun0726@163.com (J.W.);

**Keywords:** osteoinduction, hydroxyapatite/β-tricalcium phosphate, wnt signaling pathway, exercise

## Abstract

To investigate the osteoinductive mechanism triggered by hydroxyapatite/β-tricalcium phosphate (HA/β-TCP) biomaterials in mice which keep exercising. **Methods**: The HA/β-TCP biomaterials were implanted in the muscle of bilateral thighs (non-osseous sites) of eighty Balb/C mice. All animals were then randomly divided into 4 groups (n = 20). In group 1 (negative control group), the mice were fed routinely. In group 2 (running group), all mice were put on a treadmill which was set to a 60-degree incline. The mice ran 20 min thrice each day. A 5-minute break was included in the routine from day three onwards. In group 3 (weight-bearing group), all mice underwent weight-bearing running. The mice in this group performed the same routine as group 2 while carrying 5 g rubber weights. In group 4 (positive control group), dexamethasone was injected in the implanted sites of the biomaterials from the day of the operation. All mice were injected once per week and received a total of 8 injections. One and eight weeks after surgery, the blood serum was collected to detect inflammatory and immunological factors by ELISA. In addition to this, biomaterial specimens were obtained to observe inflammatory and osteogenic levels via histological staining and to facilitate analysis of the osteogenic mechanism by Western Blot. **Results**: The inflammation indexes caused by surgery were alleviated through running or weight-bearing running: The tumor necrosis factor-α (TNF-α) and interleukin-6 (IL-6) levels were significantly reduced in groups 2 and 3 at week 8. Exercise also enhanced the secretion of interferon-γ (IFN-γ) in mice; this can strengthen their immunity. The new bone tissues were observed in all groups; however, the area percentage of new bone tissues and the number of osteoblasts were highest in the weight-bearing group. Furthermore, the key proteins of wingless/integrated (Wnt) signaling pathway, Wnt1, Wnt3a, and β-catenin, were up-regulated during osteoinduction. This up-regulation activated runt-related transcription factor-2 (Runx2), increased the expression of osteopontin (OPN) and osteocalcin (OCN). **Conclusion**: Weight-bearing exercise can promote the bone and bone marrow formation through the Wnt signaling pathway: Observations documented here suggest that the proper exercise is beneficial to the recovery of bone damage.

## 1. Introduction

Common bone injuries or defects are often occasionally a difficult period of recovery for patients. For single osteoporosis-related fractures, the number of patients is forecasted to exceed 3 million per year by 2025 in the USA [1]. Therefore, the recovery of bone injuries in a short time is an urgent scientific problem. Recently, synthetic biomaterials have been identified as promising scaffolds to encourage bone repair. Scaffolds are biomaterials that are designed to properly match the natural structure and properties of bone, and they enhance all the biological processes involved in bone formation. Scaffolds are implanted in areas that require bone tissue or bone grafts due to several reasons including severe bone loss after trauma, large bone defects, tumor removal, or some reconstructive surgeries. Hydroxyapatite/β-tricalcium phosphate (HA/β-TCP) is a type of these scaffolds with excellent biocompatibility, biodegradation, osteoconductivity, and osteoinductivity [2]. An additional development in this field that supports the use of synthetic biomaterials is the emergence of an algorithm, based on mechanical biology, capable of predicting the optimal load for scaffolds: scaffolds under optimal load facilitate larger amounts of bone formation [3]. Furthermore, recent advances in the application of mesenchymal stem cells in tissue engineering, in terms of their ability to promote wound healing and tissue regeneration, provide new ways to improve complex treatments that require bone regeneration [4].

This study will investigate how exercise facilitates osteoinduction. Exercise has been proposed as a supplementary means of recovering bone health in transformational regenerative medicine. Exploratory research in this area suggests that a moderately high level of physical activity coupled with a low level of sedentary behavior could reduce the risk of chronic disease after serious orthopedic injuries [5]. However, care must be taken to avoid secondary injuries, especially in elderly patients [6]. It has been shown that physical activity could affect the immune system; the intensive exercise can lead to suppression of the activity of several kinds of immune cells, while moderate physical activity could stimulate and enhance the immunity not only in athletes but also in the general population [7,8]. Exactly how the immune system is activated by exercise remains unclear. Nevertheless, what has been proved is that calcium phosphate biomaterial could induce new bone formation in non-osseous sites of animals. In addition, exercise was able to enhance the ectopic bone formation of biomaterials in the muscles of mice through immunoregulation [9]. 

To explore how exercise facilitates osteoinduction, this study will draw focus to the Wingless/integrated (Wnt) signaling pathways. Wnt signaling pathways are evolutionarily conserved and implicated in the processes of cell fate determination, such as cell differentiation, proliferation, and apoptosis during cell development [10]. To date, three Wnt signaling pathways have been revealed, including the canonical Wnt/β-catenin pathway, the noncanonical Wnt/Ca^2+^ pathway, and the Wnt/planar cell polarity (PCP) pathway [11]. The canonical Wnt/β-catenin pathway plays an important role in the regulation of bone formation and has become a new hotspot in the field of the pathogenesis of skeletal diseases and bone metabolism [12,13]. However, little is known about whether a Wnt/β-catenin signaling pathway could promote ectopic bone formation induced by HA/β-TCP biomaterials through exercise and immunoregulation in mice. Here, we will explore the mechanism of ectopic bone formation triggered by HA/β-TCP biomaterials, and bone mass augment promoted by exercise mediated by immunoregulation. Our study will provide medical advice for patients with orthopedic injury who can use a combination therapy of rehabilitation and biological immunotherapy.

## 2. Materials and Methods

### 2.1. Materials Preparation

The cylinder-shaped HA/β-TCP biomaterial was purchased from Hunan GongChuang Biofunctional Materials Co., LTD (Hunan, China), which contained 40% HA and 60% β-TCP, the materials were scanned by scanning electron microscopy (SEM) with the machine Hitachi S-4800 (Japan) to demonstrate the microstructure of the materials. The product was sterile, vacuum-packed, and cut into (Φ3 × 5) mm shape before use.

### 2.2. Animal Surgery

**Figure 1:**
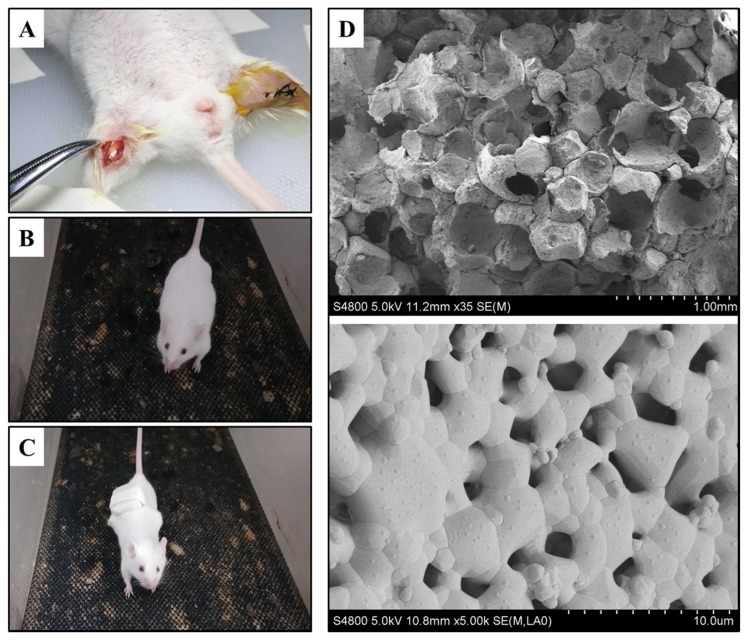
(**A**) The hydroxyapatite/β-tricalcium phosphate (HA/β-TCP) biomaterial transplanted site (left side) and suturing during surgery (right side). (**B**) Running mouse of group 2 on the treadmill. (**C**) Weight-bearing running mouse with rubber of group 3 on the treadmill. (**D**) The micropores (upper side, bar: 1 mm) and super-micropores (downside, bar: 10 μm) of HA/β-TCP biomaterials scanned by scanning electron microscopy (SEM).

Eighty Balb/C mice were obtained from Dossy Biological Technology Company (Chengdu, China). All animals were maintained in a temperature and light-controlled environment ventilated with filtered air. During the surgery, all mice were anesthetized with an intraperitoneal injection of pentobarbital sodium. The hair on the bilateral thigh was removed using an electric shaver and the skin underneath was disinfected with ethanol, and then an approximately 10-mm longitudinal skin incision was made and parallel to the femur, after that, an approximately 8-mm longitudinal muscle pouch was prepared immediately along skin incision. Next, two (Φ3 × 5) mm HA/β-TCP were implanted into the left and right thigh muscle of each mouse, respectively (Figure 1A). Finally, the wounds were closed with single interrupted suturing, and penicillin was injected intramuscularly to prevent infection.

The study has been approved by the Animal Care and Use Committee of Chengdu University. The operative procedures and animal care were performed in compliance with NIH guidelines on the care and use of laboratory animals, under the supervision of a licensed veterinarian.

### 2.3. Animal Grouping

After surgery, all mice were randomly divided into 4 groups (*n* = 20). In group 1 (negative control group), all mice were regularly fed without any treatment. In group 2 (running group), a treadmill for mice was set to a 60-degree incline, all mice were put on the treadmill to run for 20 min thrice as 6 m/h speed-moderate exercise for mice. A 5-minute break was included in the routine from day three onwards (Figure 1B). In group 3 (weight-bearing group), all mice underwent weight-bearing running. The mice in this group performed the same routine as group 2 while carrying 5 g rubber weights (Figure 1C). In group 4 (positive control group), 0.5 mL dexamethasone (0.3 mg/kg) was injected into the implant sites of biomaterials from the day of the operation once per week, a total of eight injections. All experiments were carried out for a total of eight weeks and no mice were exited due to death or illness during this period.

### 2.4. Sample Harvesting

One week and eight weeks later, the blood was collected from caudal vein and centrifuged to collect serum in all groups, respectively. Three mice were killed at week one, and 6 specimens were harvested to fix in 10% neutral formalin buffer solution for paraffin embedding in each group; then 8 weeks after surgery, the remaining seventeen animals were sacrificed, and 24 specimens were harvested to store in liquid nitrogen for Western Blot, while 10 specimens were fixed in 10% neutral formalin buffer solution for paraffin embedding in each group.

### 2.5. Enzyme-Linked Immunosorbent Assay

The levels of tumor necrosis factor-α (TNF-α), interleukin-6 (IL-6), and interferon-γ (IFN-γ) in the serum of four groups were measured by enzyme-linked immunosorbent assay (ELISA) using the respective kits and following manufacturers’ protocols (BioLegend, San Diego, CA, USA). Briefly, 96-well microplates were coated with 100 μL/well of 1 μg/mL rabbit-anti-mouse TNF-α, IL-6, and IFN-γ monoclonal antibodies in phosphate-buffered saline (PBS) and stored at 4 °C overnight. After coating, the microplates were washed five times with PBS adding Tween 20 (PBST), and then incubated with 100 μL/well of 1:20 dilution of serum samples for 2 h at 37 °C and the standards were set as control. After incubation, wells were washed five times with PBST and then incubated with 100 μL/well of 100 ng/mL biotinylated goat anti-mouse TNF-α or rabbit-anti-mouse IL-6 and IFN-γ monoclonal antibodies in PBS containing 1% BSA for 1 h at 37 °C. After that, the wells were washed another five times with PBST and then incubated with 100 μL/well of streptavidin conjugated to horseradish-peroxidase substrate for 15 min at 37 °C. The reaction was stopped by H_2_SO_4_. Finally, the measurement of OD_450_ value was performed.

### 2.6. Histological Staining

After tissue fixation, the specimens were decalcified in 10% ethylenediaminetetraacetic acid (EDTA), pH 7.0, for about 20 days at room temperature, washed with diethylpyrocarbonate (DEPC) water, dehydrated, and embedded in paraffin. The embedded specimens were cut into 5 μm thick histological sections and transferred onto 3-aminopropyrytrietoxy silane-coated glass slides. The sections of all groups were stained with hematoxylin and eosin (HE); in addition, the sections of group 3 were also stained with safranin-fast green (SFG), methylene blue-basic fuchsin solution (MBFS), and Masson-trichrome (MT) staining.

### 2.7. The Area Percentage of New Bone Tissues

Sample slides were scanned with a NanoZoomer Digital Pathology scanner (NDP, Japan), and the areas of new bone growth as well as total tissues were encircled then measured via an automatic process. Ultimately, the area percentage of new bone tissues was calculated as the ratio between the total of new bone area and the total tissue area. An osteocyte count was also taken at this time, and all osteocytes were labeled with flags.

### 2.8. Western Blot

One protein sample was extracted from five implanted biomaterial specimens of each group and bone of neonatal mouse with radio-immunoprecipitation assay (RIPA) lysis buffer. Then, the proteins were separated using a 10% polyacrylamide gel. After transferring the proteins onto a nitrocellulose membrane, the membrane was blocked with a 5% defatted milk solution and probed with mouse antibody against Wnt1 (1:1000, Proteintech, Rosemont, IL, USA), Wnt3a (1:1000, AFFINITY, Cincinnati, OH, USA), Wnt5a (1:600, Proteintech), β-catenin (1:1000, AFFINITY), runt-related transcription factor-2 (RUNX-2, 1:600, AFFINITY), osteopontin (OPN, 1:2000, Proteintech), osteocalcin (OCN, 1:1000, AFFINITY), and glyceraldehyde-3-phosphate dehydrogenase (GAPDH, 1:5000, Santa Cruz, Santa Cruz, CA, USA), and then probed with a secondary antibody using ALP conjugated anti-mouse IgG (1:5000, Santa Cruz, USA). At last, blots were developed using an ECL plus kit (GE, Boston, MA, USA). The membranes were scanned with an iBright imager (Thermo Fisher, Waltham, MA, USA) and the fluorescent signals were quantified with the ImageJ software (National Institutes of Health, Bethesd, MD, USA). The relative expression of proteins was quantitated based on band integrated optical density (IOD) value normalized to GAPDH.

### 2.9. Statistical Analysis

Data are expressed as means ± standard deviation ($\bar{x}\pm s$) and were analyzed by Student t-test (SPSS 22.0, USA). A *P* < 0.05 was considered statistically significant.

## 3. Results

### 3.1. The Characteristic of Biomaterials

The HA/β-TCP biomaterials were scanned by SEM. The scans revealed an abundance of micropores and super-micropores inside the materials, the diameter of micropores is approximately 300–500 μm, while the diameter of super-micropores is approximately 1 μm (Figure 1D). All pores are internally connected to provide nutrition for new blood vessel and bone formation.

### 3.2. Inflammation after Implantation

**Figure 2:**
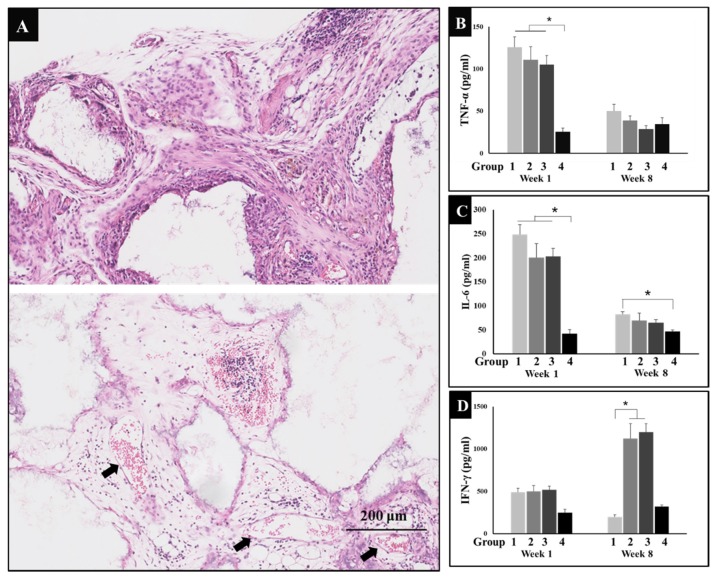
(**A**) Hematoxylin and eosin (HE) micrograph of group 3 (*n* = 6) showed inflammatory reactions and new blood vessels one week after surgery, arrow: blood vessel; bar: 200 μm. (**B**) The levels of tumor necrosis factor-α (TNF-α) secreted in serum of four groups (*n* = 5) at weeks 1 and 8; comparison among group 4 and other the three groups at week 1, * *P* < 0.05. (**C**) The levels of interleukin-6 (IL-6) secreted in serum of four groups (*n* = 5) at weeks 1 and 8; comparison among group 4 and other three groups at week 1, comparison between group 1 and group 4 at week 8, * *P* < 0.05. (**D**) The levels of interferon-γ (IFN-γ) secreted in serum of four groups (*n* = 5) at weeks 1 and 8; comparison among group 1 and groups 2, 3 at week 8, * *P* < 0.05. Group 1 = negative control group; Group 2 = running group; Group 3 = weight-bearing group; Group 4 = positive control group.

Surgery is bound to cause inflammation in both general blood circulation and local tissues. Therefore, inflammatory reactions were observed in HE sections, in addition to this, typical inflammatory factors, TNF-α and IL-6 levels in serum were detected by ELISA. HE micrograph results showed that inflammatory cells including macrophages, neutrophils, lymphocytes, etc., invaded the micropores of implanted materials forming mesenchymal tissues in the margin and central region of biomaterials, while lots of new blood vessels appeared one week after surgery (Figure 2A). In addition to this, levels of TNF-α and IL-6 sharply increased in groups 1, 2, and 3, while the positive control group (group 4) maintained the normal level, since the dexamethasone is a steroid anti-inflammatory drug which does not only promote bone formation, but also reduces the inflammation caused by surgery. After eight weeks of recovery, the inflammatory levels of TNF-α and IL-6 decreased. As indicated by the image below, it seems running can reduce inflammation to some extent (Figure 2B,C).

### 3.3. Immune Regulation through Exercise 

IFN-γ is an immune regulator that can recruit and activate macrophages. In addition, IFN-γ can activate cytotoxicity T lymphocyte (CTL) and promote B cell to produce opsonizing antibodies. Therefore, the level of IFN-γ was also determined by ELISA. The results showed that IFN-γ secretion increased significantly in groups 2 and 3 at week eight of this experiment (Figure 2D). This indicates that exercise could enhance the level of IFN-γ to regulate and heighten immunity in mice.

### 3.4. New Bone and Bone Marrow Formation

**Figure 3:**
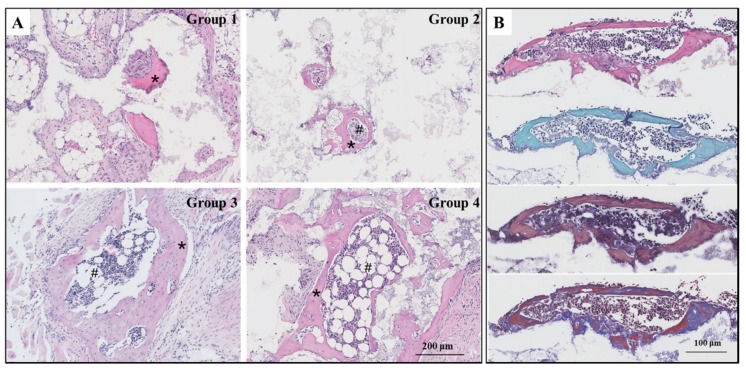
(**A**) HE micrograph showed bone and bone marrow formation induced by HA/β-TCP biomaterials in four groups (*n* = 10) in mice at week 8, *: new bone; #: new bone marrow; Group 1: negative control group; Group 2: running group; Group 3: weight-bearing group; Group 4: positive control group; bar: 200 μm. (**B**) The special staining of serial sections of group 3 (*n* = 3), HE, safranin-fast green (SFG), methylene blue-basic fuchsin solution (MBFS), and Masson-trichrome (MT) staining (from top to bottom), were conducted to identify the bone tissues, bar: 100 μm.

With or without eight weeks’ exercise or dexamethasone injection, all the biomaterials were induced ectopic bone formation. However, the amount of new bone and bone marrow tissues generated was disproportionate in the four groups. In group 1, few bone tissues were observed and no bone marrow tissues growth was evident. In groups two through four, a substantial amount of bone and bone marrow tissues developed. The distribution of bone growth here can be attributed to the various treatments each group of mice experienced running, weight-bearing running, or dexamethasone administration (Figure 3A). The special staining of serial sections, SFG, MBFS, and MT staining, were performed to identify bone tissues present in group 3. The cartilage is stained red and the bone is stained green in SFG staining. Our results show only green. This proves that osteoinduction occurred through intramembranous ossification as opposed to endochondral osteogenesis. We also observed that the osteoblasts were stained dark blue, while bone was stained red in MBFS staining. The MT staining showed that some bone tissues containing collagen fibers were stained red, indicating that the bone tissues observed were mature and able to transform collagen into calcium salt (Figure 3B).

### 3.5. The Osteoinductive Efficiency

**Figure 4:**
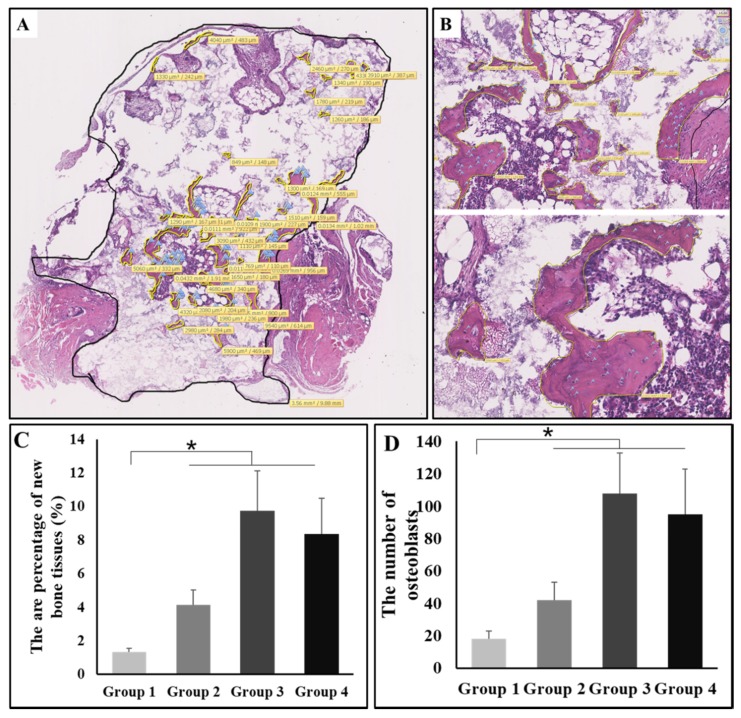
(**A**) The method about how the area percentage of new bone and the number of osteocytes were calculated. (**B**) The localized enlarged schematics of figure A. (**C**) The area percentage of new bone tissues in four groups (*n* = 10) at week 8; comparison among group 1 and other three groups, * *P* < 0.05. (**D**) The number of osteocytes in four groups (*n* = 10) at week 8; comparison among group 1 and other three groups, * *P* < 0.05. Group 1: negative control group; Group 2: running group; Group 3: weight-bearing group; Group 4: positive control group.

To quantify the effect of intervention on osteoinduction by exercise with or without weight-bearing, we measured the area percentage of new bone tissues as well as the number of osteocytes with NDP built-in function. To be specific, new bone tissues were circled with yellow lines and the area was automatically calculated and labeled in view; likewise, the total tissues were circled with a black line. The area percentage of new bone was calculated as the ratio between the total of new bone area and the total tissue area. In addition to this, the osteocytes were labeled with blue flags, and the total number of osteocytes was also counted (Figure 4A). The enlarged schematics are shown in Figure 4B. According to the statistical analysis, the area percentage of new bone was highest in group 3, and the value of groups two through four was significantly higher than that of group 1 (*P* < 0.05, Figure 4C). The number of osteocytes was also highest in group 3, and the value of groups two through four was also significantly higher than that of group 1 (*P* < 0.05, Figure 4D).

### 3.6. Wnt/β-catenin Signaling Pathway

**Figure 5:**
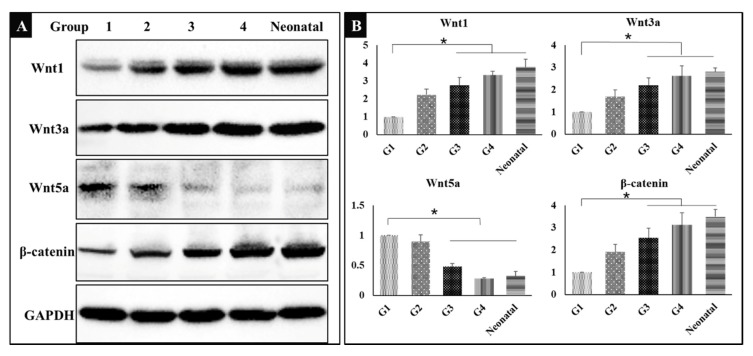
(**A**) Western blot showing the expression of wingless/integrated 1 (Wnt1), Wnt3a, Wnt5a, β-catenin, and glyceraldehyde-3-phosphate dehydrogenase (GAPDH) in five groups (*n* = 3). G1: negative control group; G2: running group; G3: weight-bearing group; G4: positive control group; Neonatal: the neonatal bone, control. (**B**) The corresponding gray-scale value of Wnt1, Wnt3a, Wnt5a and β-catenin in the five groups (*n* = 3); comparison among G1 and other three groups (G3, G4, and Neonatal), * *P* < 0.05.

It was hypothesized that bone induction was associated with the canonical Wnt signaling pathway; therefore, the related proteins were detected by Western Blot. The results showed that Wnt1, Wnt3a, and β-catenin were up-regulated to activate Wnt/β-catenin signaling pathway, while Wnt5a was down-regulated in groups 3 and 4, the bone of neonatal mice was used as control (Figure 5A). The results shown indicate that exercise or dexamethasone activated the Wnt signaling pathway to induce ectopic bone formation. The gray-scale value of each band was converted to the relative expression of these proteins (Figure 5B, * *P* < 0.05). 

### 3.7. The Expression of Osteogenesis Related Proteins

**Figure 6:**
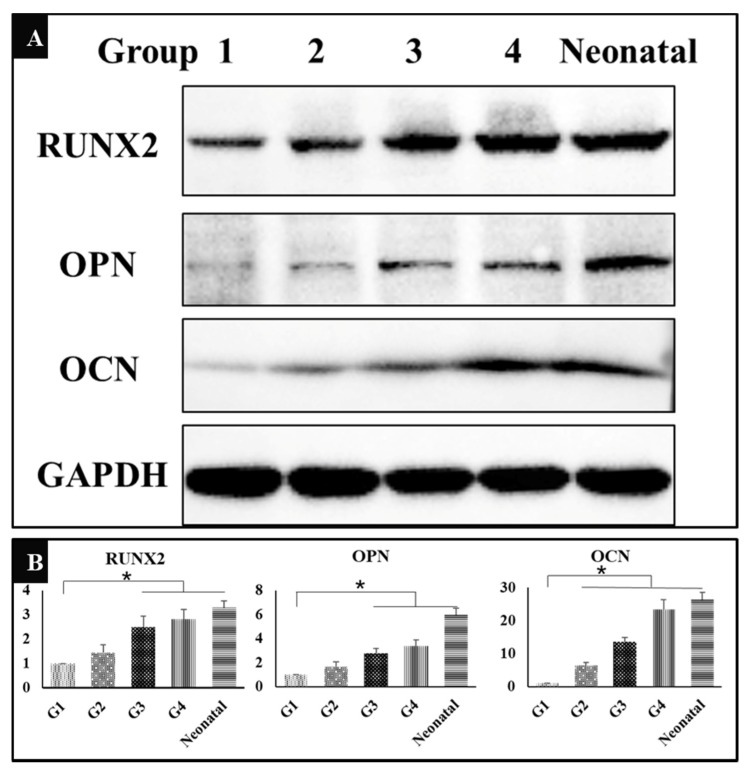
(**A**) Western Blot showing the expression of runt-related transcription factor-2 (RUNX2), osteopontin (OPN), osteocalcin (OCN), and GAPDH in five groups (*n* = 3). G1: negative control group; G2: running group; G3: weight-bearing group; G4: positive control group; Neonatal: neonatal bone, control. (**B**) The corresponding gray-scale value of RUNX2, OPN, and OCN in the five groups (*n* = 3); comparison of RUNX2 and OPN among G1 and other three groups (G3, G4, and Neonatal), comparison of OCN among G1 and other four groups (G2, G3, G4, and Neonatal), * *P* < 0.05.

In addition to identifying osteogenesis induced by HA/β-TCP from histological view, we also verified the expression of osteogenesis related proteins, Runx2, OPN, and OCN by Western Blot. The results showed that the expression of all the three proteins had been significantly up-regulated in groups 3 and 4 in contrast to group 1, while the neonatal bone was used as control (Figure 6A), indicating that both exercise and dexamethasone could enhance bone induction to stimulate the generation of new bone tissues through activation of RUNX2 pathway. The gray-scale value of each band was converted to the relative expression of these proteins (Figure 6B, * *P* < 0.05).

## 4. Discussion

The immune system includes the coordinated action of a variety of immune cells that can produce different pro- or anti-inflammatory cytokines, these include e.g., TNF-α, interleukins, and chemokines in a very complex pattern. When a bone is subjected to trauma, the numerous cytokines initiate immune reactions that eventually instigate a regenerative process. In our experimental design, the surgery of biomaterial implantation caused trauma, thereby inducing inflammatory reactions. Normal wound healing can be roughly divided into four continuous and overlapping phases: hemostasis, inflammation, proliferation, and remodeling [14]. During the inflammation phase, the platelets released chemokines for leukocytes to enhance inflammation, including IL-1α, IL-1β, IL-6, TNF-α, platelet-derived growth factor (PDGF), and transforming growth factor-β (TGF-β), etc. [15,16]. Our results show that IL-6 and TNF-α increased one week after implantation; these cytokines play an important role in initiating the chemotaxis of neutrophils, monocytes and fibroblasts [17]. These leukocytes could resolve the inflammation through removing the dead cells and recruiting endothelial cells and fibroblasts in the first two weeks [18,19,20]; however, it still could cause chronic inflammation if earlier phases of the healing process have failed to clear dead cells and debris [14,21]. In this study, there were lots of neutrophils, macrophages, fibroblasts aggregated inside and around the biomaterials, which were recruited by inflammatory factors one week after surgery, and some inflammatory cells were still observed in the histological staining eight weeks after surgery.

Osteoinduction is the induction of bone tissue production in animals: Osteoinductive biomaterials stimulate and regulate the formation of calcified bone matrix through a complex process. Research suggests that this process involves a link between inflammation and ectopic new bone formation: Activation of the canonical Wnt/β-catenin pathway and the non-canonical Wnt/PKCδ pathway is involved in inflammation-induced osteogenesis [22]. The process of osteoinduction is always accompanied by inflammation which was caused by surgical trauma, and the inflammation can activate endothelial cells and vascular endothelial growth factor (VEGF), etc., these cells and cytokines were usually used to be seed cells and osteogenic biomolecules in bone tissue engineering [23,24]. Our results show high levels of inflammation in the week following transplantation. The levels of inflammation were decreased by exercise with or without weight-bearing over time. This leads us to propose that the relative decrease of inflammatory index may impact the amount of new bone tissues during osteoinduction.

Exercise is beneficial for the improvement of immune system function, and prolonged lack of exercise can depress immunity; it also can repair the function of T cells, natural killer (NK) cells, and neutrophils, and decrease the incidence of cancer [25,26]. Therefore, proper physical activity level could be beneficial for recovering of serious orthopedic injury [5]. Our previous study revealed that exercise could activate the immunological factors that promote osteoinduction in mice [9]. The results of this study are consistent with this revelation. We observed the levels of IFN-γ one and eight weeks after surgery and found that the level of IFN-γ was significantly increased by exercise. Exercise could recruit and activate macrophages, activate CTL, and promote B cell to produce opsonizing antibodies as an immune regulator [27,28]. Our results show that the amount of new bone tissues in running groups was significantly higher than that in negative control group, indicating that there may be a relationship between osteoinduction and immunity, which will be explored in further studies. 

It is known that the TGF-β/Smads, Wnt/β-catenin, Notch, Hedgehog, and FGF signaling pathways all participate in osteoblast differentiation during bone formation [29,30,31,32]. We assumed that all of these signaling pathways might occasion synergistic action during bone development. Alternatively, we suspected that one pathway may play a major role in bone induction. As noted in our earlier work, the TGF-β/Smads signaling pathway is active in osteoinduction triggered by HA/β-TCP biomaterials [33]. This study prompted us to recognize that the Wnt/β-catenin pathway plays an important role in bone induction. The reason might be that exercise could engage the Wnt/β-catenin pathway through activation of the immune system. Therefore, osteoinduction of biomaterials can be seen as synergistic action of Wnt/β-catenin and TGF-β/Smads signaling pathways. The details and determinants of this synergistic action, including the impact of each pathway, will be explored in our future research.

## 5. Conclusions

Observations documented here suggest that exercise facilitates osteoinduction. The exploratory processes outlined above show how running, in particular weight-bearing running, can foster osteoinduction induced by calcium phosphate biomaterials in mice. The relationship observed between exercise and osteoinduction compels us to recommend that orthopedic patients engage in controlled physical activity in order to expedite recovery after bone injury: When performed under medical supervision, weight-bearing running exercises can facilitate higher osteogenic efficiency; the mechanism of ectopic bone formation is mediated by immunoregulation through the Wnt/β-catenin signaling pathway. We encourage practitioners to act on and interrogate this core insight: Exercise can be applied to facilitate osteoinduction in transformational regenerative medicine.
